# Targeting cannabinoid type 2 receptors alters human tau levels in ex vivo brain slice cultures

**DOI:** 10.1002/alz.093442

**Published:** 2025-01-03

**Authors:** Valerie L Joers, Ila Bagheri, Jazmyn Coronado, Cecilia Hillard, Malu Gámez Tansey

**Affiliations:** ^1^ University of Florida College of Medicine, Gainesville, FL USA; ^2^ Medical College of Wisconsin, Milwaukee, WI USA

## Abstract

**Background:**

The presence of Tau pathology is strongly associated with the clinical symptoms and cognitive decline found in Alzheimer’s disease (AD), suggesting that targeting pathological tau may be a more effective therapeutic approach. Microglia have been implicated in tauopathies as their activation is strongly related to the progression of tau phosphorylation and aggregation potentially due to dysfunctional lysosomal activity. Cannabinoid type 2 receptors (CB2) are highly expressed in immune cells and upregulated in activated microglia under conditions of neurologic disease, such as AD. CB2 deficient models have demonstrated a reduction in inflammation and amyloid plaque deposition, suggesting a role of CB2 on the immune system to influence AD‐related pathologies. However, there are limited findings on the CB2 regulation of tau accumulation.

**Method:**

We have generated organotypic brain slice cultures from CB2‐EGFP/f/f and CB2‐knockout mice and transduced with human wild‐type and mutant (P301L or P301L/S320F) tau viruses, previously shown to accumulate total and phosphorylated human tau and develop insoluble tau aggregates. As a complimentary approach, slices were treated with CB2‐selective ligands. Slices were evaluated for tau aggregation and microglial phenotypes using biochemical fractionation and western immunoblotting analyses.

**Result:**

In our hands, brain slice cultures overexpressing human P301L‐tau generate soluble total and phosphorylated tau species but do not produce urea‐soluble aggregates. Our preliminary work demonstrated that P301L expressing brain slice cultures deficient in CB2 do not have altered high‐salt soluble total or phosphorylated tau compared to CB2‐EGFP/f/f slices. Yet, treatment with CB2 inverse agonist SR144528 reduced phosphorylated tau (Ser202/Thr205) in the soluble fraction (p = 0.0011) in CB2‐EGFP/f/f brain slices over‐expressing P301L/S320F human tau compared to vehicle treatment. Further evaluations on tau aggregation are being conducted on brain slice cultures from CB2‐EGFP/f/f and CB2‐knockout mice overexpressing P301L/S320F tau.

**Conclusion:**

These results suggest that CB2 alters the function of immune cells to reduce tau species in a stimulation dependent‐manner. Future studies will evaluate microglia‐restricted CB2 knock‐out brain slices to advance our understanding of the role of brain CB2 in the microglial contribution to pathologic tau removal and potentially highlight CB2 as a therapeutic target against tauopathies.

